# A systematic review and narrative summary of family-based smoking cessation interventions to help adults quit smoking

**DOI:** 10.1186/s12875-016-0457-4

**Published:** 2016-06-24

**Authors:** Gill Hubbard, Trish Gorely, Gozde Ozakinci, Rob Polson, Liz Forbat

**Affiliations:** School of Health Sciences, University of Stirling, Highland Campus, Centre for Health Science, Old Perth Road, Inverness, UK; School of Medicine, Medical and Biological Sciences Building, University of St Andrews, St Andrews, UK; Highland Health Sciences Library, University of Stirling, Highland Campus, Centre for Health Science, Inverness, UK; Australian Catholic University, Canberra, Australia

**Keywords:** Smoking cessation, Family, Intervention studies, Systematic review

## Abstract

**Background:**

Smoking is the most significant preventable cause of morbidity and early mortality in the world. The family is an influential context in which smoking behaviour occurs.

**Methods:**

A systematic review and narrative summary of family-based interventions to help adults quit smoking was conducted.

**Results:**

Eight controlled trials were included. Risk of bias was high. The smoking-related outcome of the intervention was self-reported smoking status/abstinence, validated by objective measures (including saliva thiocynate or breath carbon monoxide). Follow-up ranged from 6 weeks to 5 years. The main target groups were: pregnant women (1), pregnant women who smoked (2), men at risk of cardiovascular disease (2), adult smokers (1), parents who smoked (1) and couples who both smoked (1). Interventions included family members but most did not go further by drawing on family, systemic or relational theories to harness the influence of family on smoking behaviour. Only three studies directly compared the effects on smoking behaviour of a family-based (i.e., interventions that involve a member of the family) versus an individual-based (i.e., interventions that use behaviour change techniques that focus on the individual) intervention. None of these studies found significant differences between groups on the smoking behaviour of the main target group.

**Conclusions:**

We have yet to develop family-based smoking cessation interventions that harness or re-direct the influence of family members on smoking behaviour in a positive way. Thus, it is likely that individualised-approaches to smoking cessation will prevail.

**Electronic supplementary material:**

The online version of this article (doi:10.1186/s12875-016-0457-4) contains supplementary material, which is available to authorized users.

## Background

Smoking is the most significant preventable cause of morbidity and early mortality in the world [1]. It is responsible for an estimated 6 million deaths annually [2]. Thus, helping people quit smoking is a global public health priority.

The family is an influential context in which smoking behaviour occurs [3]. For instance, parental and sibling smoking is a significant determinant of smoking uptake by children and young people [4] and cohabitants’ smoking status is a major determinant for changes in smoking behaviour among adults [5–9].

Social support is the main theoretical concept used for understanding smoking cessation in families [10, 11]. Family members’ supportive and undermining behaviours are correlated with a smoker’s likelihood of making a quit attempt and achieving abstinence [12–14]. Observational studies spanning several decades link the continuance of smoking to both the absence of positive partner support behaviours (e.g., expressing confidence in the smoker’s ability to quit) and the presence of negative partner behaviours (e.g., commenting that ‘smoking is a dirty habit’) [14, 15]. Reviews of literature suggest that interventions for smoking designed to increase social support to help adults quit smoking have been unsuccessful [11, 16–18]. A recent systematic review by Park and colleagues of 13 interventions designed to increase support from a spouse, partner, friend, or co-worker did not find greater rates of successful smoking cessation [18]. The reviewers, however, suggest that no conclusions can be made about the impact of social support on smoking cessation due to methodological limitations of the included studies as well as the likelihood that interventions did not increase the quality or quantity of partner social support [18].

The review reported in this article, addresses one of the key problems of the review by Park and colleagues, which is an assumption that social support from family is equivalent to that from friends and co-workers. A difficulty with smoking cessation interventions that harness social support to change smoking behaviour is the conflation of different sources and types of social support. These interventions invite support from spouse, intimate other, friend, relative or co-worker. The problem with this is that these relationships are different and therefore their influence on smoking behaviour also differs. A recent study about the influence of pro-smoking media (e.g., smoking in movies, advertising in magazines) on smoking in young people highlights that when participants were with friends, pro-smoking media exposures were associated with stronger smoking intentions and lower smoking refusal self-efficacy whereas these associations were not present when participants were with family [19]. A study of home smoking bans found that adolescent smoking up-take was stronger when neither parent smoked but their friends smoking behaviour did not moderate the effect of home smoking bans on adolescent smoking behaviour [20]. Consequently, the influence of family and friends on smoking behaviour is likely to differ. Family/kin has been defined as a group comprising relationships that persist over time, are emotionally intense and involve high levels of intimacy in day-to-day life [21]. Modern family structure is diverse [22] and family can refer to single and dual-parent/caregiver families, same and different sex married, civil partnership, and co-habiting couples. While many kin relations are non-voluntary, friendships (kith), in contrast, are considered profoundly voluntary and usually informal and reciprocal, based on mutual interests and social needs [23]. Neighbourly or friendship relationships foster a sense of belonging based on proximity or warmth [24], but unlike family ties, which remain fairly consistent throughout old age, contact with neighbours and friends may be subject to variation. Because they are not formally prescribed, friendships require more initiative and consequently, may decline when events such as illness or disability makes interaction difficult [25]. Relationships tend to become specialized in their provision, some of which are provided by relatives (for example, instrumental support and nurturance) and some by friends (such as social integration) [26]. Given these differences between kith and kin, it may be likely that their respective influence on smoking behaviour may also differ. This is why it is important to clearly distinguish the two sources of influence when developing smoking cessation interventions and hence our reason for conducting a review of *family*-based smoking cessation interventions.

The aim of this systematic review and narrative summary is to identify, describe, and synthesise the evidence about family-based interventions for smoking cessation. In doing so, we aim to develop understandings of family as the ‘active ingredient’ of smoking cessation interventions. In contrast to previous reviews, the focus was on family (kin) as opposed to, for instance, social support [18]. The objectives were to describe study design and methods, report intervention effects as well as to describe theories, procedures, functions and content of family-based interventions for smoking cessation. The findings will contribute towards understanding why previous family-based interventions may have limited effect and also point to ways in which future family-based smoking cessation interventions may be improved.

## Methods

### Search strategy

The PRISMA statement guided the conduct of this narrative review [27]. Studies were identified by structured database searches. All reviewers agreed which databases to search and search terms. One author (RP), who is a librarian and information specialist, searched 26 electronic databases (Cochrane Library, Campbell Library, EBSCO HOST (CINAHL, PsycINFO, Psychology and behavioural sciences collection, EconLit), Ovid Medline, Ovid HMIC, Ovid Embase, ProQuest (Applied Social Sciences Index and Abstracts, Social Services Abstracts, Sociological Abstracts, Australian Education Index, British Education Index, Education Resources Information Center), Prospero, PubMed, SCOPUS, Web of Science (Science Citations Index, Social Sciences Citation Index, Arts and Humanities Citation Index, Conference Proceedings Citation Index-Science, Conference Proceedings Citation Index- Social Science & Humanities, Book Citation Index-Science, Book Citation Index – Social Science and Humanities)) in June 2014, for trials of family-based interventions targeting smoking in adults published in English language with no date restriction. As an example, search terms used for Ovid Medline are shown in Table 1, and a full search strategy is available in Additional file 1.

**Table 1:** Ovid Medline search terms

#	Searches	Results	Search type
1	exp Smoking/or exp Smoking Cessation/	129169	Advanced
2	exp "Tobacco Use"/or "Tobacco Use Cessation"/or exp Tobacco/or exp Tobacco Products/or exp "Tobacco Use Cessation Products"/	148028	Advanced
3	1 or 2	149967	Advanced
4	exp family/	234438	Advanced
5	(grandparent: or grand-parent: or grandfather: or grand-father: or grandmother: or grand-mother:).af.	4543	Advanced
6	(partner: or husband: or wif: or wiv: or sibling: or brother: or sister: or mother: or father: or son: or daughter:).af.	659383	Advanced
7	(cousin: or uncle: or aunt:).af.	162264	Advanced
8	exp caregivers/	21234	Advanced
9	(caregiver: or care giver:).af.	39284	Advanced
10	4 or 5 or 6 or 7 or 8 or 9	986779	Advanced
11	3 and 10	13147	Advanced
12	limit 11 to english language	12148	Advanced
13	limit 12 to randomized controlled trial	446	Advanced
14	(rct: or random: trial: or random: control: trial: or random: stud: or random: control: stud:).af.	509205	Advanced
15	exp Randomized Controlled Trials as Topic/	93713	Advanced
16	(non random: stud: or nonrandom: stud: or non random: control: stud: or nonrandom: control: stud:).af.	3632	Advanced
17	(non random: trial: or nonrandom: trial: or non random: control: trial: or nonrandom: control: trial:).af.	1615	Advanced
18	(controlled before and after stud:).af.	368	Advanced
19	(controlled before and after trial:).af.	1176	Advanced
20	(quaziexper: or quazi-exper:).af.	2	Advanced
21	(quasi exper: or quasiexper:).af.	5403	Advanced
22	or/14-21	516489	Advanced
23	12 and 22	609	Advanced
24	13 or 23	609	Advanced
25	from 24 keep 1-609	609	Advanced

### Eligibility criteria and selection process

All reviewers were involved in selecting articles for inclusion. One reviewer (GH) independently screened titles/abstracts and all potentially relevant articles were obtained in full. Any articles that the reviewer was unsure about for inclusion were collectively discussed. Reviewers (LF, TG, GH, GO) assessed full articles against inclusion criteria set by all authors (Table 2). Inclusion criteria were based on language, type of study, participants, type of intervention and outcomes. Any disagreements were resolved through collective discussion.

**Table 2:** Inclusion and exclusion criteria

1. Not in English language	We excluded all papers not in English because of lack of translation facilities.
2. Type of study	Randomised controlled trials (RCTs), controlled non-randomised studies and controlled before and after studies. Comparison groups of the family intervention could be usual care, no intervention or another smoking cessation intervention. Feasibility and pilot studies were included if effects of the intervention were reported.
3. Type of intervention	Interventions promoting changes in adult tobacco use or prevention. Interventions involving at least one family member. Interventions that gave the option of including a family member or close friend/significant other were excluded. Interventions where the primary aim was to reduce exposure to secondhand smoke and place of smoking were excluded. Interventions delivered to whole-community or whole-population level interventions such as media campaigns or changes in the local environment, which included a discrete family-based intervention, were included.
4. Type of participants	The target of the intervention was an adult of any gender who smoked (18 years and over). One or more of the adult smoker’s family had to be involved in the intervention. Pregnant and non-pregnant and married and unmarried smokers were included. Interventions that targeted adults and children who smoked were included but only if outcomes of adults were reported separately and only if the intervention specifically targeted adult smoking behaviour. Interventions that only targeted children’s smoking behaviour were excluded.
5. Type of outcomes	Outcomes were the change in number of cigarettes smoked/smoking cessation of adults. Behaviours could be measured objectively (e.g., saliva) or by self-report questionnaire. If it was a multi-component intervention (e.g., family-based programme administered as part of a school-based programme to prevent smoking up-take in young people) then the effects of the family-based programme of the intervention must have been reported separately. Studies that aimed to shift location of smoking behaviour and reduce Environmental Tobacco Smoke as opposed to smoking cessation were excluded.

### Data extraction processes

Data extraction was conducted by one reviewer and then discussed and checked by at least one other reviewer. Disagreements were resolved by consensus among all of the reviewers. Data extraction forms that were used to collect data are described below.

#### Risk of bias

The Cochrane Risk of Bias tool was used to assess study bias [28]. This is a domain-based evaluation tool in which assessments of risk are made separately for selection, performance, detection, attrition and reporting bias, respectively. For each study, the six ‘risk of bias’ domains were addressed by answering a pre-specified question about the adequacy of the trial in relation to each domain, and judgment made on whether the study has high, low, or unclear risk of bias for that domain. Risk of bias was undertaken by two reviewers (TG, GO), with disagreements resolved by consensus.

#### Methods

The CONSORT 25-item checklist [29] was completed for each study. This checklist was used to report a study’s aims and objectives, methods (e.g., design, participants, interventions, outcomes, sample size), randomization and statistical methods, participants and numbers analysed, results of analysis and discussion (e.g., limitations, generalizability and interpretation).

#### Intervention description

To describe the interventions, a Template for Intervention Description and Replication (TIDieR) [30] was completed for each study. If studies provided a rationale for a family-based approach to help smokers to quit this was reported under the section ‘Why’; the materials and procedures used with family members were reported under the section ‘What’; the professional delivering the intervention to family members was reported under the section ‘Who provided’ and any training provided for the deliverer was also recorded; where, when and how long the intervention was delivered were reported under the sections ‘Where’ and ‘How much,’ respectively; any ‘tailoring’ or ‘modifications’ to the intervention were reported under these sections; family member adherence was reported under the section ‘How well.’ We did not describe the individualised intervention components (e.g., materials and procedures etc.) used with the participant that was making the smoking quit attempt but only those aspects of the intervention that involved a family member. This is because the focus of this review is on family (e.g., training family members to assist their partner to make a quit attempt) as opposed to individualised (e.g., goal-setting such as setting a quit date) ‘active ingredients’ of smoking cessation interventions that involve family members. In addition, if the intervention included other behaviours (e.g., diet), these were also not described.

#### Intervention function

We used a function checklist to categorise intervention functions [31]. Only the intervention functions for family members are reported for the reason described above. The function checklist designates nine functions. Five functions can be conceptualised as individual level functions and are: education (increasing knowledge or understanding), persuasion (using communication to induce positive or negative feelings or stimulate action), incentivisation (creating expectation of reward), training (imparting skills), and enablement (increasing means/reducing barriers to increase capability or opportunity) [31]. The other four intervention functions are: coercion (creating expectation of punishment or cost), restriction (using rules to reduce the opportunity to engage in the target behaviour), environmental restructuring (changing the physical or social context), and modeling (providing an example for people to aspire to or imitate), which place more emphasis on external influences and less on personal agency [31]. More than one function could be selected, for example, if the intervention involved informing the family member about the harms of smoking, this was recorded under the function ‘education’ and ‘persuasion.’

#### Theory coding scheme

The Theory Coding Scheme [32] was used to describe the theoretical basis of interventions. The Theory Coding Scheme comprises 19 items and a clear description of how to code each item. All items are listed under the following six categories, which can be used to assess the use of theory: 1) Is theory/model mentioned? 2) Are the relevant theoretical constructs targeted? 3) Is theory used to select recipients or tailored interventions? 4) Are the relevant theoretical constructs measured? 5) Is theory tested? 6) Is theory refined? Again, only theories relating to the family components of the interventions were recorded. Using this coding scheme provided a method for the systematic appraisal of family-based theoretical components of interventions as well as more general behaviour change theories and models.

### Narrative synthesis of results

The data extraction forms described above were also used to assess if it was viable to conduct a quantitative synthesis. Using Cochrane Review guidance [28], a meta-analysis was ruled out because although most studies shared a common primary outcome (smoking cessation measured by self-report and objectively), as we describe in detail below, comparison groups (interventions versus controls), participants and key components of the interventions were not the same across studies.

Synthesis involves the collation, combination and summary of the findings of individual studies included in the systematic review [33]. Narrative synthesis comprises identifying patterns, similarities and differences about the interventions and methods reported in the included studies. Four reviewers conducted the narrative synthesis (GH, LF, GO, TG) and was carried in four stages. First, articles were divided among the reviewers who extracted data from articles about each individual trial using the data extraction forms described above. All collected data were visually presented in tabular format. Second, the data extraction forms were used to produce a narrative descriptive summary of all trials methods and bias (TG/GO), rationale (GH), theories (LF), procedures (GH), functions (GH), and content (GH). These are reported in the results section below. Third, the reviewers collectively identified and discussed patterns between intervention characteristics (e.g., rationale, theories, procedures, functions and content) and the effect of the intervention on smoking cessation. The extent to which these characteristics might explain variation in the size/direction of effect was discussed. Fourth, an overall assessment of the strengths and limitations of the evidence-base about family-based approaches to help adults quit smoking were discussed by all reviewers and summarised. The outcomes of these synthesis procedures are presented in the discussion section.

## Results

**Fig. 1:**
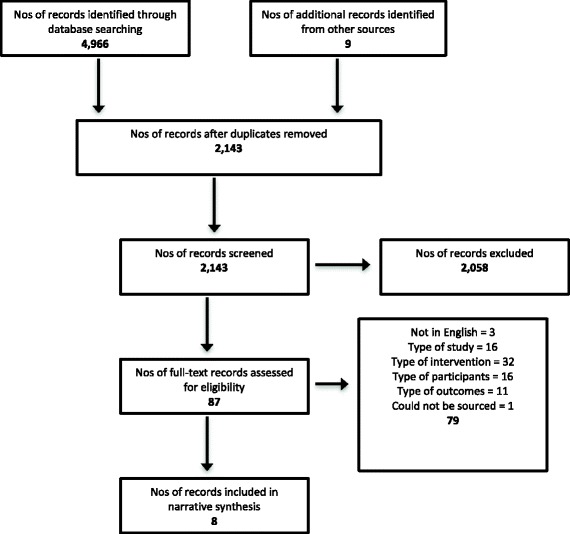
Flowchart

Figure 1 shows the flow of studies through the review process and reasons for exclusion. Searches identified 4966 potentially relevant articles, which was reduced to 2143 articles after removing duplicates. Following review of titles and abstracts, 76 full text articles were retrieved. An additional 11 articles were identified through reference lists of included articles. Of the 87 articles, eight met the selection criteria. Four studies were conducted in North America and four in European countries.

### Risk of bias

The results of the ‘risk of bias assessment’ are presented in Table 3. We judged all trials to be at unclear or high risk of bias in the majority of domains. Random sequence generation, analytic blinding and selective reporting were all identified as high risk or unclear in the majority of studies.

**Table 3:** Risk of bias

	Design and description of comparison groups	Selection bias		Performance bias	Detection bias	Attrition bias	Reporting bias	
Authors		Random sequence generation	Allocation concealment	Blinding participants/personnel	Blinding outcome assessment	Incomplete outcome data	Selective reporting	Other bias
Hjermann et al. [38]	RCT – lifestyle intervention; anti-smoking advice given individually to all smokers vs. usual care	Unclear	Low risk	High risk	Unclear	Low risk	Unclear	Unclear
McBride et al. [34]	3-group randomised controlled intervention; pregnant women; – Usual care (advice to quit and self-help guide), woman only group (usual care plus late-pregnancy relapse prevention kit and 6 counselling calls), Partner-assisted group (woman only group intervention plus their partners received telephone counselling and a support guide)	Unclear	Unclear	High risk	Unclear	Low risk	Unclear	Unclear
McIntyre-Kingsolver et al. [39]	RCT - a multi-component cognitive-behavioral smoking program vs. the same program with spouses attending and receiving training designed to increase spouse social support	High risk	Unclear	High risk	Unclear	High risk	Unclear	High risk
Nyborg and Nevid [41]	3-group, randomly assigned; effort only control (written materials), therapist administered treatment (couples received weekly counselling sessions), self-administered/minimal contact control (behavioural treatment manual and weekly telephone contact)	Unclear	Unclear	High risk	Unclear	Unclear	Unclear	Unclear
Øien et al. [36]	Controlled, prospective, intervention study of two cohorts - a prenatal, structured, multi-disciplinary smoking cessation programme vs. common, nationwide recommended, advice on lifestyle, including smoking behaviour	N/A – not RCT	N/A	High risk	Unclear	Unclear	Unclear	High risk
Patten et al. [40]	Pilot, feasibility RCT - a web-based support skills training vs. health education	Unclear	Unclear	High risk	Unclear	Low risk	Low risk	Unclear
de Vries et al. [35]	Cluster RCT - Midwives in the experimental group provided brief health counseling, self-help materials on smoking cessation during pregnancy and early postpartum, and a partner booklet. Controls received routine care	Low risk	Unclear	High risk	Unclear	Low risk	Unclear	High risk
Wood et al. [37]	Cluster RCT – a nurse-coordinated multidisciplinary, family-based preventive cardiology programme vs. usual care	Unclear	Unclear	High risk	Unclear	High risk	Low risk	Low risk

### Primary outcome and target group

The primary target group for smoking behaviour change varied across studies (Table 4); three targeted pregnant women (two of these targeted pregnant women who smoked) [34–36], two targeted men at risk of cardiovascular disease [37, 38], one study targeted adult smokers [39], and one targeted parents who smoked [40]. Only one study targeted couples who both smoked [41].

**Table 4:** Methods and results of included studies

Author	Country	Main target group for smoking cessation	Family member involved in intervention	Smoking behaviour of main target group and family member	Sample*	Reason for involving family	Smoking outcome measure/length follow up	Comparison groups (interventions described fully in Table 5)	Results: Are family-based interventions more effective?
Hjermann et al. [38]	Norway	Men at risk of coronary heart disease and aged 20-49 years with no evidence of diseases of the cardiovascular system, diabetes psychopathological disease or alcoholism	Wives	Smoking behaviour of the main target group (men at risk of coronary heart disease) and family members was not an eligibility criteria	1232 healthy, normotensive men at high risk of coronary heart disease; 604 intervention and 628 control group respectively. Number of family members not given	None given	Self-reported smoking habits; 5 years	Lifestyle intervention involving wives vs. control group (not described)	It is unclear if the ‘active ingredient’ of the wives’ involvement influenced effectiveness because this was not tested The intervention was effective for men. Tobacco consumption (expressed as number of cigarettes per man per day; pipe smoking is included taking one pack of pipe tobacco weekly to equal 7 cigarettes daily) fell about 45 % more in the intervention group than in the controls
McBride et al. [34]	North America	Pregnant women (current or recent quitters) living intimately with their partners	Partners	The main target group was a current smoker or recent quitter; smoking behaviour of partner was not included in eligibility criteria	583 pregnant women and 583 partners. 198 pregnant women in usual care group, 192 pregnant women in women only group and 193 pregnant women in the partner assisted group	Marital theory and empirical research show how marital relationships might affect provision of support for smoking cessation	Self-reported smoking status baseline (about 11 weeks of pregnancy), at 28 weeks of pregnancy, and at 2-, 6-, and 12-months postpartum. Saliva samples were collected by mail at 28 weeks of pregnancy and at 12 months postpartum from women and partners who reported not smoking	3 groups: Women in the usual care group received advice to quit and a self-help guide vs. women in the women only group also receiving a late-pregnancy relapse prevention kit (booklet and gift items) and six counseling calls vs. women in the partner-assisted group also having their partners receiving telephone counseling and a support guide emphasizing skills to help the woman build and maintain her confidence to quit smoking	No for pregnant women. Intent-to-treat analyses showed no significant differences by group in women’s reports of abstinence at any follow-up Yes for partners. In late pregnancy, more partners were abstinent in the partner assisted group (15 %) than in the usual care group (5 %), *p* = 0.02
McIntyre-Kingsolver et al. [39]	North America	Adult smoker in a committed live-in relationship with a spouse or spouse-equivalent	Spouse or partners	The main target group was a smoker; smoking behaviour of partner was not included in eligibility criteria	64 couples. Subjects were required to be in a committed, live-in relationship with a spouse or spouse-equivalent who was willing to attend the treatment sessions	Perceived helpfulness from a spouse and verbal encouragement and cooperative participation may be an asset to cessation and maintenance	Self-report smoking status and abstinence and reports of significant others; saliva thiocyanate (SCN) and/or level of alveolar carbon monoxide (CO); 1 and 6 months follow up	Spouse training vs. usual treatment to aid a smoking cessation	No. There was a consistent trend in favour of the partner training treatment, but even the largest difference (72.7 % vs. 48.4 % abstinent), at the end of treatment, was not significant.
Nyborg and Nevid [41]	North America	Couples who both smoke and live together and both seeking to quit or reduce smoking and both smoking > 20 cigarettes a day	Spouse or partner who also smoked	To be eligible both individuals that comprised the couple had to smoke	40 couples living together randomly assigned to 1 of 5 treatment groups	Social support	Self-reported abstinence post-treatment and 3 and 6 months	5 different types of smoking cessation interventions compared: 2 couple-based and 2 individual-based therapy groups and a group just given written materials	No. Abstinence rates for couples were not significantly different across groups at follow-up intervals,
Øien et al. [36]	Norway	Pregnant women	Spouse or partners	The smoking behaviour of the main target group (pregnant women) and the family member (partner) was not included in the eligibility criteria	Pregnant women and partners: intervention cohort (*N* = 2051) and the control cohort (*N* = 1788). Number of partners not given	None given	Self-reported smoking behaviour; 9-12 weeks gestation, and at 6 weeks after delivery	A cohort given smoking cessation intervention vs. a cohort not given the intervention	It is unclear if the ‘active ingredient’ of partner spouses’/partners’ involvement influenced effectiveness because this was not tested The intervention was not effective for pregnant women. Data stratified according to smoking behaviour at the beginning of pregnancy demonstrated that in the intervention cohort only one in four of the smoking women continued to smoke from the beginning of pregnancy until inclusion, with no significant difference between the cohorts. Yes for partners. In contrast, most men continued to smoke in the same period, but significantly fewer in the intervention cohort
Patten et al. [40]	North America	Parent (biological, adopted, step parent or adult guardian) who currently smoked ≥5 cigarettes per day	Child aged 13-19 years, never smoked or if a former smoker had not smoked during past 6 months, and interested in helping parent quit	The main target group (parent) was a current smoker and the family member (child) either never smoked or had quit	40 non-smoking adolescents (13–19 years) interested in helping a parent (biological, adopted, step parent or adult guardian) to quit who currently a) smoked >=5 cigarettes per day	Adolescents are concerned about parents who smoke and wish to help them quit	At each follow-up point prevalence of abstinence defined as no cigarettes smoked (not even a puff) for previous 7 days. Confirmed at 6 months by salivary cotinine concentration of <15 ng/ml. Quit attempts since time of enrolment assessed at each follow-up; 6 and 12 weeks and 6 months	2 smoking cessation interventions compared: Health education vs. support training	It is unclear if the ‘active ingredient’ of child involvement influenced effectiveness because this was not tested. The study included two different family-based interventions and about half of parents in each group reported a quit attempt since study enrolment
de Vries et al. [35]	Netherlands	Pregnant women who had been pregnant more than twice (because assumed that these women would be very unlikely to change their smoking behaviour) and smoked at least 1 cigarette a day	Partners who smoked were involved, otherwise partners not involved	The main target group (pregnant women) was a current smoker. If their partner also smoked then they were included in the intervention	141 and 177 pregnant women in intervention and control groups completed first questionnaires, respectively. Number of partners not given	None given	Self-reported: 7-day abstinence, Continuous abstinence (6 weeks postpartum), Partner smoking; Measures at pre-test and 6 weeks post-intervention and 6 weeks postpartum. Urine-cotinine levels measured by gas chromatography/mass spectrometry in sub-sample	Brief health counseling, self-help materials on smoking cessation during pregnancy and early postpartum, and a partner booklet vs. usual care and a general folder from the Dutch Smoking and Health Foundation	It is unclear if the ‘active ingredient’ of partner spouses’/partners’ involvement influenced effectiveness because this was not tested The intervention was effective for pregnant women. Significant differences were found between the two groups. Nineteen percent of the experimental group reported 7-day abstinence compared to 7 % of the control group at first follow up, and 21 and 12 %, respectively, at second follow up. For continuous abstinence these percentages were 12 % in the experimental group and 3 % in the control group The intervention was not effective for partners. No significant differences between the two groups were found for partners
Wood et al. [37]	European countries: France, Italy, Poland, Spain, Sweden, UK, Denmark, Italy,Poland, Spain, the Netherlands	Patients at least 50 years of age and less than 80 years old, with no history of cardiovascular disease but at risk of coronary heart disease with no history of severe heart failure, severe physical disability, or dementia and their partners	Spouse or partners	Smoking behaviour of the main target group (men at risk of coronary heart disease) and family members was not an eligibility criteria	1589 and 1499 patients with coronary heart disease in hospitals and 1189 and 1128 at high risk were assigned to intervention and usual care groups. 860 patients and 410 partners participated in hospital intervention programme; 947 high-risk patients and 204 partners participated in general practice intervention programme	Provide support	Self-reported cessation of smoking, validated by a breath carbon monoxide concentration of less than 6 parts per million; 12 months follow up	2 lifestyle intervention groups (hospital and general practice groups) vs. usual care (not described)	It is unclear if the ‘active ingredient’ of partner spouses’/partners’ involvement influenced effectiveness because this was not tested The intervention was effective for patients Among patients with coronary heart disease who reported smoking in the month before their cardiac event, a higher proportion in the intervention group were not smokers at 1 year compared with the usual-care group (for example, hospital intervention vs. usual care was 58 % vs. 47 % *p*=0.06) The intervention was not effective for partners. Non-smoking at 1 year was greater, although not significantly so, in the partners of patients in the intervention groups than in usual-care groups

*Studies vary in how sample size is reported and we have used available information about adult smokers (target) and family members involved

The smoking-related outcome in all studies was self-reported smoking abstinence (see Table 4). Self-reports were validated by objective measures (e.g., saliva thiocynate or breath carbon monoxide) in at least a sub-sample in five studies [34, 35, 37, 39, 40]. Follow-up varied from 6 weeks to 5 years; this represents a weakness of the shorter durations being unable to report comparable duration of cessation for comparison. Seven studies included spouse or partners [35–39, 41].

### Intervention description

Table 5 describes key intervention components, with a particular focus on family-based components. Six were smoking behaviour only interventions [34–36, 39–41] and two were multi-component lifestyle interventions [37, 38]. Four studies involved relatives in group-based sessions [35, 38–40]; four delivered family- or couple-based counselling, training and/or advice [34, 36, 37, 41]; one study provided written materials only to relatives (e.g., booklet, manual, guide, sheet) [35]. Health professionals (midwife, nurse, health advisor, primary care professionals) or behaviour change counsellors, delivered the interventions. Two studies mentioned that training was provided to the intervention deliverers [36, 40]. Intervention duration ranged from 5 weeks to 9 months. Two studies specified where the intervention was delivered, and both were in healthcare settings [36, 37]. No studies reported tailoring or modifying the intervention. Only one study measured the extent to which relatives were involved in the intervention [40].

**Table 5:** Intervention description

Author	Behaviours	Materials and procedures	Intervention function(s)	Deliverers	Duration	Tailoring	Family involvement
Hjermann et al. [38]	Smoking, diet	The wives of the subjects were invited in groups of 30-40 together with their husbands for diet and smoking information.	Education	Not described	Not described	No	Not measured
McBride et al. [34]	Smoking	6 counseling telephone calls (three in pregnancy and three in postpartum) using motivational interviewing techniques. An “It Takes Two” booklet and companion video were developed to guide couples in discussing support behaviors related to the woman’s smoking.	Education and training	Health advisor	Not described	No	Not measured
McIntyre-Kingsolver et al. [39]	Smoking	Spouse training Common examples of helpful or unhelpful behaviors were pointed out and the group was also asked to contribute examples from their own experience. Guided group discussions and direct instruction were used to try and increase positive or decrease negative spouse behaviors. Spouses were encouraged at all stages to help problem solve difficult situations (e.g., quit day) and to reward subjects for making small steps in changing their habit. It was emphasized that the posttreatment support and assistance that spouses provided was crucial to the success of the subject. Subjects were also encouraged to reward their spouses for participating in the program and for helping them.	Education and training	Counsellors were two clinical psychology graduate students with experience of conducting smoking cessation groups.	Six weekly two-hour groups sessions	No	Not measured
Nyborg and Nevid [41]	Smoking	Couples received additional written materials which provided instructions in providing mutual support for smoking reduction and cessation) The techniques included mutual modeling of appropriate nonsmoking behavior in smoking-related contexts (e.g., talking on the telephone without smoking), mutual monitoring (systematically counting each other's cigarettes), partner reinforcement for habit change, and couple reinforcement contingent upon achievement of mutual goals in changing smoking habits (e.g., the couple selects a shared reward for mutual abstinence during a predetermined period of time). Couples receiving therapist-administered treatment reviewed their progress in implementing these mutual support strategies and received therapist feedback in their treatment sessions. Weekly telephone contact was maintained with minimal contact couples during which partners reported on each other's progress and received therapist feedback.	Education and training	Behaviour therapists	8 weeks	No	Not reported
Øien et al. [36]	Smoking	Women were invited to bring their partners to the individual consultations, and if he was a smoker they were encouraged to make a smoking cessation effort together	Enablement	Primary care professionals: GPs and midwives, public health nurses. Offered a 3 h course to improve smoking cessation counselling skills	8 to 10 prenatal consultations in primary care	No	Not measured
Patten et al. [40]	Smoking	Health education control group 11 page booklet: (1) education on the health effects of smoking, (2) nicotine dependence and withdrawal symptoms, (3) readiness to quit, (4) basic communication skills between the adolescent and parent, (5) strategies on how to approach the parent about their smoking and quitting, (6) strategies on how to elicit from the parent the pros and cons of continued smoking and quitting, (7) triggers for relapse. 5 web-based group sessions for Support Skills Training: (1) rationale for treatment (i.e., Raise awareness of possible personal benefits of treatment) (e.g., dealing with anger or distress regarding parent's smoking behavior) – “You can't control your parent, only yourself. It is important to focus on what you can do as a support person”, setting S.M.A.R.T. goals and use of self-rewards and education on nicotine dependence; (2) provide education on motivation and readiness to quit; (3) using positive behaviors and statements to encourage their parent to move forward in the quitting process; (4) provide education on how smokers quit (i.e., setting a quit date, nicotine dependence treatments, coping with triggers, and social support); (5) provide education on lapse, relapse and how to reinforce (shape) progress made by the smoker and goal setting after the program ends.Web-based message board to post questions	Education and training and persuasion	6 research counsellors with Masters or Bachelors degree in behavioural health or social science. Training provided to deliver the intervention	5 weeks × 1 session × 30 min	No	95 % (19/20) adolescents completed all sessions and 79 % read the booklet
de Vries et al. [35]	Smoking	Because pregnant women motivated to quit smoking encounter difficulties to quit in the presence of a smoking partner a booklet was made for partners who also smoked	Education	Midwife	Not described	No	Not measured
Wood et al. [37]	Smoking, diet, exercise	Couples attended lifestyle assessment and group workshop about lifestyle risk factors for coronary heart disease and cardiovascular risks. Patients were provided with a personal record card for lifestyle and risk factor targets and their families with family support packs.	Education	Nurse	8 weekly sessions in hospital or general practice	No	Not measured

### Intervention function

Table 5 describes the intervention function for the family component of the intervention. Interventions could be categorised as having more than one function. An intervention function in all but one study was categorised as ‘education.’ The only study that was not categorised as ‘education’ was categorised as ‘enablement’ because couples made a quit attempt together [36]. An intervention function in four studies was categorised as ‘training’ (i.e., giving guidance and instruction to family members on how to be supportive to their relative who is making a quit attempt) [34, 39–41]. An intervention function in one study was categorised as ‘persuasion’ because the intervention emphasised the harmful effects of smoking to the child to motivate them to support their parent to quit smoking [40].

### Theoretical models

Table 6 summarises the underpinning theoretical approaches adopted in each of the studies. Where theories were described, this was often not in depth, and referred to social support or social influence theories. Only one study [34] explicitly referenced family theories such as marital theories, systemic theories or relational theories, and most papers did not reflect on or adapt their theoretical model in the light of the findings. Thus, studies in this review at best under-reported and at worse, under-theorised the models on which involvement of family was predicated.

**Table 6:** Theoretical models informing the interventions

Study	Is theory/model mentioned?	Are the relevant theoretical constructs targeted?	Is theory used to select recipients or tailored interventions?	Are the relevant theoretical constructs measured?	Is family-related theory tested?	Is theory refined?
Hjermann et al. [38]	Not reported	Not reported	Not reported	Not reported	Not reported	Not reported
McBride et al. [34]	Social support and marital theory is referred to.	Yes. Intervention objectives were to (1) encourage couple communication about helpful and unhelpful support behaviors, (2) assist partners in developing alternatives to negative behaviors, (3) prompt couples to make plans for handling high-risk situations, and (4) when appropriate, encourage and assist partner smoking cessation.An “It Takes Two” booklet and companion video were developed to guide couples in discussing support behaviors related to the woman’s smoking.	No	Yes. Partner Interaction Questionnaire to assess positive and negative perceived and provided support for cessation.	Intervention impact on support was measured. Women in all 3 groups consistently reported a decline in positive partner support from baseline to 12-month Postpartum, negative support decreased through pregnancy, but increased postpartum. Partners reported little change in positive and negative smoking-specific support that they gave in the same time frame.	Not reported
McIntyre-Kingsolver et al. [39]	Social support is cited as the driving theory, referring to a previous study which found that perceived helpfulness from a spouse during treatment was significantly related to smokers achieving and maintaining abstinence.	Yes. Common examples of helpful or unhelpful behaviors were discussed. Guided group discussions and direct instruction were used to try and increase positive or decrease negative spouse behaviors. Spouses were encouraged at all stages to help problem solve difficult situations (e.g., quit day) and to reward subjects for making small steps in changing their habit. It was emphasized that the post-treatment support and assistance that spouses provided was crucial to the success of the subject. Subjects were also encouraged to reward their spouses for participating in the program and for helping them.	Relatives are guided on how to be more/less supportive.	Partner Interaction Questionnaire measured the impact of the spouse-training treatment component. This 61-item tool taps into a variety of smoking-related spousal interactions.	Influence of social support is measured and found to not be related to self-reported smoking status at follow-up.	Not reported
Nyborg and Nevid [41]	Social support	Yes. Couples received additional written materials which provided instructions in providing mutual support for smoking reduction and cessation) The techniques included mutual modeling of appropriate nonsmoking behavior in smoking-related contexts (e.g., talking on the telephone without smoking), mutual monitoring (systematically counting each other's cigarettes), partner reinforcement for habit change, and couple reinforcement contingent upon achievement of mutual goals in changing smoking habits (e.g., the couple selects a shared reward for mutual abstinence during a predetermined period of time). Couples receiving therapist-administered treatment reviewed their progress in implementing these mutual support strategies and received therapist feedback in their treatment sessions. Weekly telephone contact was maintained with minimal contact couples during which partners reported on each other's progress and received therapist feedback.	No	Not reported	Not reported	Not reported
Øien et al. [36]	Not reported	Not reported	Not reported	Not reported	Not reported	Not reported
Patten et al. [40]	No explicit theory provided, but the link between adolescents influencing parental smoking is proposed as promoting health and reducing second-hand smoke exposure.	Not reported	Not reported	Not reported	Not reported	Not reported
de Vries et al. [35]	Theory of behaviour change, based on communication techniques, and the “health communication persuasion matrix”, based on social influence theory and self-efficacy. The authors note that reviews on smoking and pregnant women suggest to include partner smoking in programs since smoking status of the partner is a chief predictor of postpartum relapse	The intervention was focused on pregnant women. A booklet was provided to smoking fathers, encouraging cessation and support to their partner.	The booklet was given to women with smoking partners.	No, but partner smoking was measured.	Not reported	Not reported
Wood et al. [37]	Not reported	Not reported	Not reported	Not reported	Not reported	Not reported

### Effect on smoking behaviour

Table 4 shows the results of the studies. Most studies did not assess the influence of family involvement in the intervention on smoking behaviour because there was no direct comparison of a family-based smoking cessation intervention with an individualised- based smoking cessation intervention [35–38, 40]. Hence, in most studies it is not possible to determine if family was the ‘active ingredient’ or if other behaviour change active ingredients influenced intervention effectiveness. Three studies [34, 39, 41] however, do contribute towards developing an understanding of the influence of family involvement in smoking cessation interventions on smoking behaviour by comparing an intervention that involved family members with an intervention that did not included family members.

One study that targeted pregnant women who were current smokers or recent quitters and their intimate partners compared three groups: women in the usual care group received advice to quit and a self-help guide versus women in the women only group also receiving a late-pregnancy relapse prevention kit (booklet and gift items) and six counseling calls versus women in the partner-assisted group also having their partners receiving telephone counseling and a support guide emphasizing skills to help the woman build and maintain her confidence to quit smoking [34]. The study found no significant differences by group in women’s reports of abstinence at any follow-up but found that more partners were abstinent in the partner assisted group (15 %) than in the usual care group (5 %), *p* = 0.02 [34]. Another study that targeted adult smokers compared two groups: spouse training versus usual treatment to aid a smoking cessation [39]. The results show a consistent trend in favour of the partner training treatment, but even the largest difference (72.7 % vs. 48.4 % abstinent), at the end of treatment, was not significant [39]. The only study that targeted couples who smoked compared five different types of interventions: two couple-based and two individual-based therapy groups and a group just given written materials [41]. The study found that abstinence rates for couples were not significantly different across groups at follow-up.

## Discussion

Very few RCTs of smoking cessation interventions that involve family members have been conducted. Studies were too dissimilar to conduct a meaningful meta-analysis; nevertheless, our narrative synthesis of the evidence about family-based interventions for smoking cessation enables us to make a number of observations.

First, it is not possible to determine if family is a critical ‘active ingredient’ in smoking cessation interventions primarily because most studies did not include a direct comparison of a family-based smoking cessation intervention with an individualised- based smoking cessation intervention. This represents a major limitation of family-based smoking cessation intervention studies because it means that it is not possible to determine if family is the ‘active ingredient.’ Of the three studies that did directly compare the effects on smoking behaviour of a family-based (i.e., interventions that involve a member of the family) versus an individual-based (i.e., interventions that use behaviour change techniques that focus on the individual such as, setting a date for a quit attempt) intervention, the evidence suggests that family is not a key ‘active ingredient’. Although we know from epidemiological studies that family members influence smoking behaviour of other family members [4–9], we have yet to develop family-based smoking cessation interventions that harness or re-direct that influence in a positive way. Thus, it is likely that individualised-approaches to smoking cessation will prevail.

Second, we found no trials of family-based smoking interventions (defined as those targeting adult smokers and involving at least one relative) conducted outside North America and Europe. This impairs our understandings about smoking cessation interventions given that family systems vary [42] and so we might expect components of family-based smoking cessation interventions in different countries to reflect these differences.

Third, there is lack of clarity about reasons for the involvement of family members in smoking cessation interventions. Some of the interventions used social support or social influence theories to aid cessation but many did not provide a coherent rationale for adopting a family-based approach. In addition, our review highlights methodological and reporting limitations of the majority of the studies, which means that the effect of family-based smoking interventions remains uncertain. It is thus premature to draw definitive conclusions about the effect of family-based interventions to help people quit smoking.

These findings are perhaps not surprising. In the past, family-based social support interventions to help adults quit smoking have also shown disappointing results, leading those who had developed these interventions in the 1980s and 1990s to conclude that modifying longstanding interpersonal relationships to impact smoking cessation is extremely difficult [43, 44]. However, a review of these interventions suggest that their failure may reflect methodological and theoretical limitations and in particular, over-reliance on a social support model where spouses/partners learn, practice and apply various coping, problem-solving or support skills to augment smoking cessation [15]. Rohrbaugh and colleagues [15] proposed instead the use of a systemic/interactional framework where family members are not merely ‘adjunct therapists’ but are perceived as full participants with a stake in the changes that will occur. A key aim of the systemic approach advocated by Rohrbaugh and colleagues [15] is to help couples negotiate relational functions that smoking serves such as, regulating emotional expression and interpersonal closeness (e.g., smoking may convey messages such as ‘let’s relax together’). The reviewers developed FAMCON, which is based on family-systems principles and in a study involving 20 couples in which one partner (the primary smoker) continued to smoke despite having or being at significant risk for heart or lung disease. The study found that 50 % rate of stable abstinence was achieved by primary smokers over at least 6 months, which exceeds benchmark success rates reported in the literature for other comparably intensive interventions. The findings suggest that a couple-focused intervention different in concept and format from social support interventions tested in the past may hold promise for health-compromised smokers [45]. FAMCON however, is not included in this review because it did not include a control group.

Fourth, our review highlights lack of conceptual clarity about family and how it differs to other relationships such as friendships. It remains unclear what the active ingredients of family are that make it such an important context for patterns of smoking behaviour and smoking cessation efforts. Much health research located within the social sciences tends to define family loosely, often allowing participants to self-determine who and what constitutes family. McDaniel and colleagues [46] for instance, define family as “any group of people related either biologically, emotionally or legally, i.e., the group of people that the patient defines as significant for his/her wellbeing”(p2). This conceptual flexibility reflects the constructed and culturally bound nature of social relations and influence. Some studies propose the notion of a “psychological family” where the family is defined as those members who are psychologically connected [47], including “friends-like family” (p283). These social definitions stand in contrast to biomedical conceptualisations of families as sharing genetic or environmental effects, and reflect the interest in relational influence and support, rather than biological drivers. Nevertheless, unless interventionists are clear why they are proposing a family-based approach then procedures, function, and content of intervention delivery are likely to under-utilise what it is about family that makes it such a potent system for promoting behaviour change.

### Limitations

For this review we comprehensively searched a number of databases, however, we did not search for non-English publications or unpublished literature. Further, researchers do not always distinguish between, or clarify whether it is a family member or friend etc. who provides support, which increases the risk of not identifying some studies eligible for inclusion. It is possible that we missed relevant studies, although we believe that this is unlikely given our systematic search strategy. Only one reviewer screened titles/abstracts and so may have missed some studies. However, the number of retrieved articles is similar to other searches for social support smoking cessations interventions [18], suggesting that the risk of over-looking studies was minimal. Due to study resource constraints additional systematic searches for articles have not been conducted since the original search in 2014. However, PsychInfo, Ovid Embase and Ovid Medline auto-alerts suggest that no additional studies have been published. The search identified a heterogeneous range of studies (intervention approaches, targets, focus on reduction or cessation, contexts and methodologies), which precluded the ability to see the identified articles as a cohesive group. Further, family members were not treated similarly across studies, with some research positioning relatives as supportive and others as a simultaneous target of smoking behavior change. The heterogeneity and small number of articles also prevented the conduct of a meta-analysis of study findings.

## Conclusion

It is premature to conclude that family-based interventions are not an effective component to cessation programmes. Current evidence suggests that family-based interventions are inadequately theorized. This is in sharp contrast to complex intervention guidelines, which note that using theory is important [48]. Our review indicates that family-based approaches, which rely exclusively on social support or social influence models to aid a quit attempt are missing a trick; future family-based interventions should draw on systemic and relational theories which offer more fine-grained understandings of the mutuality of relational influence, current family context and the genealogy of familial health behaviours. That is, social support theory while having a façade of utility, does not offer a framework for examining the rich current and past influences of familial relationships on health behaviours. Practitioners in primary care have a unique opportunity to make use of social support theories and systemic relational theories where they may have contact with multiple people within one family system.

## Additional file

Additional file 1: Searches conducted. (DOC 167 kb)
