# Supplementary material for: A systematic review and narrative summary of family-based smoking cessation interventions to help adults quit smoking
Source: BMC Fam Pract. 2016 Jun 24;17:73. doi: 10.1186/s12875-016-0457-4 (PMC4921023; doi:10.1186/s12875-016-0457-4)
Supplement: Additional file 1: — Searches conducted. (DOC 167 kb) [file 12875_2016_457_MOESM1_ESM.doc]

**Search Strategies**

**Cochrane**

Search Name:

Date Run: 01/05/14 12:05:13.756

Description:

ID Search Hits

#1 MeSH descriptor: [Tobacco] explode all trees 139

#2 MeSH descriptor: [Smoking] explode all trees 5431

#3 MeSH descriptor: [Smoking Cessation] explode all trees 3034

#4 #1 or #2 or #3 6919

#5 MeSH descriptor: [Family] explode all trees 5569

#6 #4 and #5 166

#7 MeSH descriptor: [Caregivers] explode all trees 1202

#8 #4 and #7 8

#9 #8 not #6 6

**Ovid Medline/PubMed**

| Ovid MEDLINE(R) 1946 to Present with Daily Update | | | |
| --- | --- | --- | --- |
| **#** | **Searches** | **Results** | **Search Type** |
| 1 | exp Smoking/ or exp Smoking Cessation/ | 129169 | Advanced |
| 2 | exp "Tobacco Use"/ or "Tobacco Use Cessation"/ or exp Tobacco/ or exp Tobacco Products/ or exp "Tobacco Use Cessation Products"/ | 148028 | Advanced |
| 3 | 1 or 2 | 149967 | Advanced |
| 4 | exp family/ | 234438 | Advanced |
| 5 | (grandparent: or grand-parent: or grandfather: or grand-father: or grandmother: or grand-mother:).af. | 4543 | Advanced |
| 6 | (partner: or husband: or wif: or wiv: or sibling: or brother: or sister: or mother: or father: or son: or daughter:).af. | 659383 | Advanced |
| 7 | (cousin: or uncle: or aunt:).af. | 162264 | Advanced |
| 8 | exp caregivers/ | 21234 | Advanced |
| 9 | (caregiver: or care giver:).af. | 39284 | Advanced |
| 10 | 4 or 5 or 6 or 7 or 8 or 9 | 986779 | Advanced |
| 11 | 3 and 10 | 13147 | Advanced |
| 12 | limit 11 to english language | 12148 | Advanced |
| 13 | limit 12 to randomized controlled trial | 446 | Advanced |
| 14 | (rct: or random: trial: or random: control: trial: or random: stud: or random: control: stud:).af. | 509205 | Advanced |
| 15 | exp Randomized Controlled Trials as Topic/ | 93713 | Advanced |
| 16 | (non random: stud: or nonrandom: stud: or non random: control: stud: or nonrandom: control: stud:).af. | 3632 | Advanced |
| 17 | (non random: trial: or nonrandom: trial: or non random: control: trial: or nonrandom: control: trial:).af. | 1615 | Advanced |
| 18 | (controlled before and after stud:).af. | 368 | Advanced |
| 19 | (controlled before and after trial:).af. | 1176 | Advanced |
| 20 | (quaziexper: or quazi-exper:).af. | 2 | Advanced |
| 21 | (quasi exper: or quasiexper:).af. | 5403 | Advanced |
| 22 | or/14-21 | 516489 | Advanced |
| 23 | 12 and 22 | 609 | Advanced |
| 24 | 13 or 23 | 609 | Advanced |
| 25 | from 24 keep 1-609 | 609 | Advanced |

**OVID Embase**

| **Embase** 1974 to 2014 May 13 | | | |
| --- | --- | --- | --- |
| **#** | **Searches** | **Results** | **Search Type** |
| 1 | exp smoking/ or exp smoking regulation/ or exp parental smoking/ or exp smoking ban/ or exp smoking cessation program/ or exp maternal smoking/ or exp smoking cessation/ or exp smoking habit/ or exp "smoking and smoking related phenomena"/ or paternal smoking/ | 224158 | Advanced |
| 2 | exp tobacco smoke/ or exp tobacco/ or exp tobacco dependence/ or exp "tobacco use"/ or exp tobacco consumption/ | 226961 | Advanced |
| 3 | exp Smoking/ or exp Smoking Cessation/ | 212253 | Advanced |
| 4 | exp "Tobacco Use"/ or "Tobacco Use Cessation"/ or exp Tobacco/ or exp Tobacco Products/ or exp "Tobacco Use Cessation Products"/ | 235884 | Advanced |
| 5 | or/1-4 | 251343 | Advanced |
| 6 | exp family/ | 332109 | Advanced |
| 7 | (grandparent: or grand-parent: or grandfather: or grand-father: or grandmother: or grand-mother:).af. | 6309 | Advanced |
| 8 | (partner: or husband: or wif: or wiv: or sibling: or brother: or sister: or mother: or father: or son: or daughter:).af. | 1080988 | Advanced |
| 9 | (cousin: or uncle: or aunt:).af. | 221153 | Advanced |
| 10 | exp caregivers/ | 39084 | Advanced |
| 11 | (caregiver: or care giver:).af. | 58318 | Advanced |
| 12 | or/6-11 | 1516120 | Advanced |
| 13 | 5 and 12 | 27008 | Advanced |
| 14 | limit 13 to english language | 25342 | Advanced |
| 15 | limit 14 to (randomized controlled trial or controlled clinical trial) | 685 | Advanced |
| 16 | exp "randomized controlled trial (topic)"/ | 51257 | Advanced |
| 17 | (rct: or random: trial: or random: control: trial: or random: stud: or random: control: stud:).af. | 503393 | Advanced |
| 18 | (non random: stud: or nonrandom: stud: or non random: control: stud: or nonrandom: control: stud:).af. | 4961 | Advanced |
| 19 | (non random: trial: or nonrandom: trial: or non random: control: trial: or nonrandom: control: trial:).af. | 2234 | Advanced |
| 20 | (controlled before and after stud:).af. | 387 | Advanced |
| 21 | (controlled before and after trial:).af. | 34 | Advanced |
| 22 | (quaziexper: or quazi-exper:).af. | 4 | Advanced |
| 23 | (quasi exper: or quasiexper:).af. | 7628 | Advanced |
| 24 | or/16-23 | 512437 | Advanced |
| 25 | 14 and 24 | 973 | Advanced |
| 26 | 15 or 25 | 1108 | Advanced |

**CINAHL**

|  | Tuesday, May 20, 2014 10:39:20 AM |
| --- | --- |

| **#** | **Query** | **Limiters/Expanders** | **Last Run Via** | **Results** |
| --- | --- | --- | --- | --- |
| S21 | S15 OR S20 | Search modes - Boolean/Phrase | Interface - EBSCOhost Research Databases Search Screen - Basic Search Database - CINAHL Plus with Full Text | 92 |
| S20 | S16 OR S19 | Search modes - Boolean/Phrase | Interface - EBSCOhost Research Databases Search Screen - Basic Search Database - CINAHL Plus with Full Text | 54 |
| S19 | S17 AND S18 | Search modes - Boolean/Phrase | Interface - EBSCOhost Research Databases Search Screen - Basic Search Database - CINAHL Plus with Full Text | 54 |
| S18 | metaanal* or meta anal* | Search modes - Boolean/Phrase | Interface - EBSCOhost Research Databases Search Screen - Basic Search Database - CINAHL Plus with Full Text | 27,518 |
| S17 | s3 and s10 | Search modes - Boolean/Phrase | Interface - EBSCOhost Research Databases Search Screen - Basic Search Database - CINAHL Plus with Full Text | 4,019 |
| S16 | s3 and s10 | Limiters - Publication Type: Meta Analysis Search modes - Boolean/Phrase | Interface - EBSCOhost Research Databases Search Screen - Basic Search Database - CINAHL Plus with Full Text | 20 |
| S15 | S11 OR S14 | Search modes - Boolean/Phrase | Interface - EBSCOhost Research Databases Search Screen - Basic Search Database - CINAHL Plus with Full Text | 72 |
| S14 | S12 AND S13 | Search modes - Boolean/Phrase | Interface - EBSCOhost Research Databases Search Screen - Basic Search Database - CINAHL Plus with Full Text | 56 |
| S13 | systemat* review* | Search modes - Boolean/Phrase | Interface - EBSCOhost Research Databases Search Screen - Basic Search Database - CINAHL Plus with Full Text | 42,990 |
| S12 | s3 and s10 | Search modes - Boolean/Phrase | Interface - EBSCOhost Research Databases Search Screen - Basic Search Database - CINAHL Plus with Full Text | 4,019 |
| S11 | S3 AND S10 | Limiters - Publication Type: Systematic Review Search modes - Boolean/Phrase | Interface - EBSCOhost Research Databases Search Screen - Basic Search Database - CINAHL Plus with Full Text | 67 |
| S10 | S4 OR S5 OR S6 OR S7 OR S8 OR S9 | Search modes - Boolean/Phrase | Interface - EBSCOhost Research Databases Search Screen - Basic Search Database - CINAHL Plus with Full Text | 253,544 |
| S9 | caregiver* or care giver* | Search modes - Boolean/Phrase | Interface - EBSCOhost Research Databases Search Screen - Basic Search Database - CINAHL Plus with Full Text | 35,465 |
| S8 | (MH "Caregivers") | Search modes - Boolean/Phrase | Interface - EBSCOhost Research Databases Search Screen - Basic Search Database - CINAHL Plus with Full Text | 19,455 |
| S7 | cousin* or uncle* or aunt* | Search modes - Boolean/Phrase | Interface - EBSCOhost Research Databases Search Screen - Basic Search Database - CINAHL Plus with Full Text | 15,388 |
| S6 | partner* or husband* or wif* or wiv* or sibling* or brother* or sister* or mother* or father* or son* or daughter* | Search modes - Boolean/Phrase | Interface - EBSCOhost Research Databases Search Screen - Basic Search Database - CINAHL Plus with Full Text | 122,077 |
| S5 | grandparent* or grand-parent* or grandfather* or grand-father* or grandmother* or grand-mother* | Search modes - Boolean/Phrase | Interface - EBSCOhost Research Databases Search Screen - Basic Search Database - CINAHL Plus with Full Text | 2,224 |
| S4 | (MH "Family+") OR (MH "Family Relations+") OR (MH "Family Attitudes+") | Search modes - Boolean/Phrase | Interface - EBSCOhost Research Databases Search Screen - Basic Search Database - CINAHL Plus with Full Text | 141,454 |
| S3 | S1 OR S2 | Search modes - Boolean/Phrase | Interface - EBSCOhost Research Databases Search Screen - Basic Search Database - CINAHL Plus with Full Text | 46,349 |
| S2 | (MH "Tobacco+") OR (MH "Tobacco Abuse Control (Saba CCC)") OR (MH "Tobacco Abuse (Saba CCC)") | Search modes - Boolean/Phrase | Interface - EBSCOhost Research Databases Search Screen - Basic Search Database - CINAHL Plus with Full Text | 5,549 |
| S1 | (MH "Smoking+") OR (MH "Smoking Cessation Programs") OR (MH "Smoking Cessation Assistance (Iowa NIC)") OR (MH "Smoking Cessation") | Search modes - Boolean/Phrase | Interface - EBSCOhost Research Databases Search Screen - Basic Search Database - CINAHL Plus with Full Text | 44,064 |

**HMIC i**

| **HMIC Health Management Information Consortium** 1979 to March 2014 | | | |
| --- | --- | --- | --- |
| **#** | **Searches** | **Results** | **Search Type** |
| 1 | exp Smoking control/ or exp Smoking policy/ or exp Smoking/ or exp Anti smoking campaigns/ or exp Passive smoking/ or exp Smoking treatment/ or exp Smoking cessation/ | 4985 | Advanced |
| 2 | exp Tobacco/ or exp Tobacco consumption/ or exp Tobacco smoke/ or exp Tobacco products/ or exp Tobacco substitutes/ | 1119 | Advanced |
| 3 | (tobac: or smok: or cigar:).af. | 8949 | Advanced |
| 4 | exp Families/ | 3197 | Advanced |
| 5 | (grandparent: or grand-parent: or grandfather: or grand-father: or grandmother: or grand-mother:).af. | 122 | Advanced |
| 6 | (partner: or husband: or wif: or wiv: or sibling: or brother: or sister: or mother: or father: or son: or daughter:).af. | 17337 | Advanced |
| 7 | (cousin: or uncle: or aunt:).af. | 1507 | Advanced |
| 8 | (caregiver: or care giver:).af. | 1116 | Advanced |
| 9 | exp carers/ | 3905 | Advanced |
| 10 | or/1-3 | 8950 | Advanced |
| 11 | or/4-9 | 25279 | Advanced |
| 12 | 10 and 11 | 766 | Advanced |
| 13 | exp Randomised controlled trials/ | 2180 | Advanced |
| 14 | (rct: or random: trial: or random: control: trial: or random: stud: or random: control: stud:).af. | 5195 | Advanced |
| 15 | (non random: stud: or nonrandom: stud: or non random: control: stud: or nonrandom: control: stud:).af. | 58 | Advanced |
| 16 | (non random: trial: or nonrandom: trial: or non random: control: trial: or nonrandom: control: trial:).af. | 51 | Advanced |
| 17 | (controlled before and after stud:).af. | 41 | Advanced |
| 18 | (controlled before and after trial:).af. | 2 | Advanced |
| 19 | (quaziexper: or quazi-exper:).af. | 0 | Advanced |
| 20 | (quasi exper: or quasiexper:).af. | 349 | Advanced |
| 21 | or/13-20 | 5525 | Advanced |
| 22 | 12 and 21 | 33 | Advanced |

**HMIC ii**

| |  | [**# ▲**](http://ovidsp.uk.ovid.com/sp-3.12.0b/ovidweb.cgi?&S=APIMPDJJFAHFDIGAFNMKGBOFFJOCAA00&Sort+Sets=descending) | **Searches** | **Results** | **Search Type** | | --- | --- | --- | --- | --- | |  | 1 | exp Smoking control/ or exp Smoking policy/ or exp Smoking/ or exp Anti smoking campaigns/ or exp Passive smoking/ or exp Smoking treatment/ or exp Smoking cessation/ | 4985 | Advanced | |  | 2 | exp Tobacco/ or exp Tobacco consumption/ or exp Tobacco smoke/ or exp Tobacco products/ or exp Tobacco substitutes/ | 1119 | Advanced | |  | 3 | (tobac: or smok: or cigar:).af. | 8949 | Advanced | |  | 4 | exp Families/ | 3197 | Advanced | |  | 5 | (grandparent: or grand-parent: or grandfather: or grand-father: or grandmother: or grand-mother:).af. | 122 | Advanced | |  | 6 | (partner: or husband: or wif: or wiv: or sibling: or brother: or sister: or mother: or father: or son: or daughter:).af. | 17337 | Advanced | |  | 7 | (cousin: or uncle: or aunt:).af. | 1507 | Advanced | |  | 8 | (caregiver: or care giver:).af. | 1116 | Advanced | |  | 9 | exp carers/ | 3905 | Advanced | |  | 10 | or/1-3 | 8950 | Advanced | |  | 11 | or/4-9 | 25279 | Advanced | |  | 12 | 10 and 11 | 766 | Advanced | |  | 13 | exp systematic reviews/ | 2390 | Advanced | |  | 14 | systematic: review:.af. | 3756 | Advanced | |  | 15 | exp meta analysis/ | 653 | Advanced | |  | 16 | (metaanal: or meta anal:).af. | 1524 | Advanced | |  | 17 | or/13-16 | 4470 | Advanced | |  | 18 | 12 and 17 | 28 | Advanced | |
| --- | --- | --- | --- | --- | --- | --- | --- | --- | --- | --- | --- | --- | --- | --- | --- | --- | --- | --- | --- | --- | --- | --- | --- | --- | --- | --- | --- | --- | --- | --- | --- | --- | --- | --- | --- | --- | --- | --- | --- | --- | --- | --- | --- | --- | --- | --- | --- | --- | --- | --- | --- | --- | --- | --- | --- | --- | --- | --- | --- | --- | --- | --- | --- | --- | --- | --- | --- | --- | --- | --- | --- | --- | --- | --- | --- | --- | --- | --- | --- | --- | --- | --- | --- | --- | --- | --- | --- | --- | --- | --- | --- | --- | --- | --- | --- |

**Campbell Library/Prospero**

(tobac* OR smok* OR cigar*) AND (family OR families OR grandparent* OR grand-parent* OR grandfather* OR grand-father* OR grandmother* OR grand-mother*) OR (partner* OR husband* OR wif* OR wiv* OR sibling* OR brother* OR sister* OR mother* OR father* OR son* OR daughter* OR spous*) OR (cousin* OR uncle* OR aunt*) OR (carer* OR caregiver* OR care-giver*)

**Applied Social Sciences Index and Abstracts/Social Services Abstracts/ECONLit/Australian Education Index**

(systematic* review* OR metaanal* OR meta-anal*) and (tobac* or smok* or cigar*) AND (family OR families OR grandparent* OR grand-parent* OR grandfather* OR grand-father* OR grandmother* OR grand-mother* OR partner* or husband* or wif* or wiv* or sibling* or brother* or sister* or mother* or father* or son* or daughter* or spous* OR cousin* or uncle* or aunt* or carer* or caregiver* or care-giver*)

**SCOPUS/Web of Knowledge**

SR/MA strategy

(((TITLE-ABS-KEY(tobac* OR smok* OR cigar*)) AND ((TITLE-ABS-KEY(family OR families OR grandparent* OR grand-parent* OR grandfather* OR grand-father* OR grandmother* OR grand-mother*)) OR (TITLE-ABS-KEY(partner* OR husband* OR wif* OR wiv* OR sibling* OR brother* OR sister* OR mother* OR father* OR son* OR daughter* OR spous*)) OR (TITLE-ABS-KEY(cousin* OR uncle* OR aunt*)) OR (TITLE-ABS-KEY(carer* OR caregiver* OR care-giver*)))) AND (TITLE-ABS-KEY(systematic* review* OR metaanal* OR meta-anal*))) AND (cess* OR stop* OR ceas*)

RCT strategy:

(((TITLE-ABS-KEY(tobac* OR smok* OR cigar*)) AND ((TITLE-ABS-KEY(family OR families OR grandparent* OR grand-parent* OR grandfather* OR grand-father* OR grandmother* OR grand-mother*)) OR (TITLE-ABS-KEY(partner* OR husband* OR wif* OR wiv* OR sibling* OR brother* OR sister* OR mother* OR father* OR son* OR daughter* OR spous*)) OR (TITLE-ABS-KEY(cousin* OR uncle* OR aunt*)) OR (TITLE-ABS-KEY(carer* OR caregiver* OR care-giver*)))) AND ((TITLE-ABS-KEY(rct* OR random* trial* OR random* control* trial* OR random* stud* OR random* control* stud*)) OR (TITLE-ABS-KEY(nonrandom* stud* OR non-random* stud* OR non random* control* stud* OR nonrandom* control* stud*)) OR (TITLE-ABS-KEY(nonrandom* trial* OR non-random* trial* OR non random* control* trial* OR nonrandom* control* trial*)) OR (TITLE-ABS-KEY(nonrandom* trial* OR non-random* trial* OR non random* control* trial* OR nonrandom* control* trial*)) OR (TITLE-ABS-KEY("controlled before and after stud*")) OR (TITLE-ABS-KEY("controlled before and after trial*")) OR (TITLE-ABS-KEY(quaziexper* OR quazi-exper*)) OR #)) AND (cess* OR stop* OR ceas*)

**British Education Index/Australian Education Index/Educational Resources Information Center**

(systematic* review* OR metaanal* OR meta-anal*) AND (tobac* OR smok* OR cigar*) AND (family OR families OR grandparent* OR grand-parent* OR grandfather* OR grand-father* OR grandmother* OR grand-mother* OR partner* OR husband* OR wif* OR wiv* OR sibling* OR brother* OR sister* OR mother* OR father* OR son* OR daughter* OR spous* OR cousin* OR uncle* OR aunt* OR carer* OR caregiver* OR care-giver*)

And

(rct* or random* trial* or random* control* trial* or random* stud* or random* control* stud* OR nonrandom* stud* or non-random* stud* or non random* control* stud* or nonrandom* control* stud* OR nonrandom* trial* or non-random* trial* or non random* control* trial* or nonrandom* control* trial* OR controlled before and after stud* OR controlled before and after trial* OR quaziexper* or quazi-exper* OR quasiexper* or quasi-exper*) AND (tobac* OR smok* OR cigar*) AND (family OR families OR grandparent* OR grand-parent* OR grandfather* OR grand-father* OR grandmother* OR grand-mother* OR partner* OR husband* OR wif* OR wiv* OR sibling* OR brother* OR sister* OR mother* OR father* OR son* OR daughter* OR spous* OR cousin* OR uncle* OR aunt* OR carer* OR caregiver* OR care-giver*)

**PsychINFO, Psychology and Behavioural Sciences Collection**

|  | Friday, June 27, 2014 6:02:01 AM |
| --- | --- |

| **#** | **Query** | **Limiters/Expanders** | **Last Run Via** | **Results** | **Action** |
| --- | --- | --- | --- | --- | --- |
| S11 | S8 AND S10 | Search modes - Boolean/Phrase | Interface - EBSCOhost Research Databases Search Screen - Advanced Search Database - PsycINFO;Psychology and Behavioral Sciences Collection | 166 |  |
| S10 | S5 AND S9 | Search modes - Boolean/Phrase | Interface - EBSCOhost Research Databases Search Screen - Advanced Search Database - PsycINFO;Psychology and Behavioral Sciences Collection | 11,758 |  |
| S9 | S1 OR S2 OR S3 OR S4 OR S6 OR S7 | Search modes - Boolean/Phrase | Interface - EBSCOhost Research Databases Search Screen - Advanced Search Database - PsycINFO;Psychology and Behavioral Sciences Collection | 758,360 |  |
| S8 | systematic* review* OR ( metaanal* OR meta anal* ) | Search modes - Boolean/Phrase | Interface - EBSCOhost Research Databases Search Screen - Advanced Search Database - PsycINFO;Psychology and Behavioral Sciences Collection | 34,385 |  |
| S7 | carer* | Search modes - Boolean/Phrase | Interface - EBSCOhost Research Databases Search Screen - Advanced Search Database - PsycINFO;Psychology and Behavioral Sciences Collection | 10,716 |  |
| S6 | famil* | Search modes - Boolean/Phrase | Interface - EBSCOhost Research Databases Search Screen - Advanced Search Database - PsycINFO;Psychology and Behavioral Sciences Collection | 486,756 |  |
| S5 | tobac* or smok* or cigar* | Search modes - Boolean/Phrase | Interface - EBSCOhost Research Databases Search Screen - Advanced Search Database - PsycINFO;Psychology and Behavioral Sciences Collection | 59,592 |  |
| S4 | grandparent* or grand-parent* or grandfather* or grand-father* or grandmother* or grand-mother* | Search modes - Boolean/Phrase | Interface - EBSCOhost Research Databases Search Screen - Advanced Search Database - PsycINFO;Psychology and Behavioral Sciences Collection | 5,614 |  |
| S3 | partner* or husband* or wif* or wiv* or sibling* or brother* or sister* or mother* or father* or son* or daughter* | Search modes - Boolean/Phrase | Interface - EBSCOhost Research Databases Search Screen - Advanced Search Database - PsycINFO;Psychology and Behavioral Sciences Collection | 323,621 |  |
| S2 | cousin* or uncle* or aunt* | Search modes - Boolean/Phrase | Interface - EBSCOhost Research Databases Search Screen - Advanced Search Database - PsycINFO;Psychology and Behavioral Sciences Collection | 31,651 |  |
| S1 | caregiver* or care giver* | Search modes - Boolean/Phrase | Interface - EBSCOhost Research Databases Search Screen - Advanced Search Database - PsycINFO;Psychology and Behavioral Sciences Collection | 42,201 |  |
